# APOE3 Christchurch is associated with sphingolipids recycling and glial lipid remodeling in autosomal dominant Alzheimer’s disease

**DOI:** 10.21203/rs.3.rs-10297182/v1

**Published:** 2026-07-15

**Authors:** Iván Daniel Salomón-Cruz, Sara Camila Agudelo-Castrillon, Juan Pablo Barbosa-Carvajal, Laura Alejandra Lozano-Trujillo, Mateo Hoyos Ríos, Lina M. Trujillo-Chacón, Andrés Villegas, Edison Osorio, Geysson Javier Fernandez, Estela Area-Gómez, Gloria Patricia Cardona-Gómez

**Affiliations:** University of Antioquia; University of Antioquia; University of Antioquia; University of Antioquia; University of Antioquia; University of Antioquia; University of Antioquia; University of Antioquia; University of Antioquia; Centro de Investigaciones Biológicas Margarita Salas; University of Antioquia

**Keywords:** Post-mortem brain, Alzheimer’s disease, neutral lipids, phospholipids, sphingolipids, gangliosides, salvage pathway

## Abstract

Alzheimer’s disease is characterized by profound disturbances in brain lipid metabolism, which regulate membrane integrity, connectivity, immune response, and cell survival. However, the mechanisms by which the protective APOE3 Christchurch variant modulates lipid homeostasis in autosomal dominant AD remain poorly understood. Here, we investigated lipid changes in postmortem brains carriers of PSEN1-E280A mutation, including APOE3Ch variant. Using a multimodal approach integrating thin-layer chromatography lipid profiling, enzymatic activity assays, digital PCR, immunofluorescence, flow cytometry, and single-nucleus RNA sequencing, we characterized lipid composition and transcriptional expression in the cerebral cortex. Familial and sporadic AD brains exhibited extensive remodeling of lipid pathways, including depletion of structural phospholipids and marked alterations in sphingolipid metabolism. Notably, APOE3Ch carriers displayed reduced cholesterol and phospholipid content, preservation of ceramide pools, and enrichment of specific ganglioside fractions, accompanied by increased sphingomyelinase activity and coordinated downregulation of genes involved in sphingolipid biosynthesis and remodeling. Single-nucleus transcriptomic analyses further revealed cell-type-specific alterations across glial populations, including reduced pruning of differentiated oligodendrocytes and suppression of lipid metabolic process in astrocytes and microglia. Together, these findings suggest that APOE3Ch promotes a reduced de novo biosynthesis of cholesterol and a distinct sphingolipid metabolic state characterized by enhanced lipid recycling, potentially attenuating lipid-driven neuroinflammatory responses.

## INTRODUCTION

Alzheimer’s disease (AD) is the leading cause of dementia worldwide and represents an escalating global health challenge. Neuropathologically, AD is characterized by extracellular amyloid-β (Aβ) plaque deposition, intracellular neurofibrillary tangles composed of hyperphosphorylated tau, chronic neuroinflammation, and progressive synaptic and neuronal loss (1–3). Although the amyloid cascade hypothesis has historically dominated the conceptual framework of AD pathogenesis, increasing evidence indicates that disturbances in systemic and cerebral lipid metabolism are integral drivers of disease progression rather than secondary consequences of neurodegeneration (4–6). Lipids constitute nearly half of the brain dry weight and are essential determinants of membrane architecture, myelin stability, vesicular trafficking, mitochondrial integrity, and synaptic signaling (7). Consequently, disruptions in lipid homeostasis can profoundly influence multiple neuropathological processes involved in AD (8, 9).

Among the lipid classes implicated in AD, cholesterol metabolism occupies a central role. Altered cerebral cholesterol redistribution affects amyloid precursor protein (APP) processing, membrane raft organization, and synaptic plasticity (10–12). These abnormalities are tightly linked to apolipoprotein E (APOE), particularly the APOE4 allele, the strongest genetic risk factor for sporadic AD, which disrupts physiological lipid transport and accelerates neurodegenerative processes (13). In parallel, disturbances in glycerolipid metabolism, including triacylglycerol accumulation and lipid droplet formation, reflect altered cellular energetics and metabolic stress within reactive brain tissue. Structural phospholipids, especially phosphatidylcholine and phosphatidylethanolamine species enriched in docosahexaenoic acid (DHA), are consistently depleted in plasma, cerebrospinal fluid, and brain tissue from individuals with mild cognitive impairment (MCI) and AD, supporting their relevance as early biomarkers of disease progression (14–16).

Sphingolipid metabolism has additionally emerged as a critical regulatory axis linking membrane structure to bioactive signaling and neuroimmune responses. Ceramides, sphingomyelins, glycosphingolipids, and gangliosides are fundamental constituents of neuronal and myelin membranes and participate in the regulation of the “sphingolipid rheostat,” a dynamic balance among sphingomyelin, ceramide, sphingosine, and sphingosine-1-phosphate (S1P) that determines cell survival or death (17, 18). In AD, shifts toward ceramide accumulation and reduced S1P signaling promote oxidative stress, apoptosis, mitochondrial dysfunction, and chronic neuroinflammation (19, 20).

Importantly, lipid dysregulation in AD is highly compartmentalized across glial populations and vulnerable brain regions. Astrocytes are the principal regulators of cerebral cholesterol synthesis and lipid redistribution through APOE-containing lipoproteins (21, 22). Microglia coordinate immune surveillance and lipid clearance but acquire a dysfunctional, pro-inflammatory phenotype associated with intracellular lipid droplet accumulation during aging and AD (12, 20). Oligodendrocytes similarly depend on tightly regulated synthesis of cholesterol and sphingolipids to maintain myelin integrity and axonal support (23, 24). These observations highlight the importance of spatially resolved lipidomic approaches to understand how glial-specific metabolic programs contribute to neurodegeneration or resilience.

Genetic evidence further supports a causal relationship between lipid homeostasis and AD susceptibility. APOE is the major genetic modifier of late-onset AD through its central role in lipid transport and metabolic regulation within the brain (22, 25). Whereas APOE4 markedly accelerates neuropathology, rare APOE variants can confer protection. Notably, the APOE3 Christchurch variant (APOE3Ch; R154S) delays cognitive decline for decades in carriers from the Colombian PSEN1-E280A familial AD kindred despite extensive amyloid pathology (26–28). Because PSEN1-E280A mutation carriers exhibit a highly predictable disease trajectory (29, 30), this cohort provides a unique opportunity to investigate early metabolic alterations preceding clinical symptoms. Our previous studies presented that systemic lipidomic remodeling begins during childhood and adolescence in mutation carriers (31). However, whether these early peripheral alterations persist as region-specific and cell-specific lipidomic signatures within the postmortem human brain, and how protective APOE variants modulate glial lipid pathways, remains poorly understood.

Here, we demonstrate that APOE3Ch carriers exhibit a distinct spatial lipidomic and transcriptional profile characterized by reduced cholesterol and phospholipid content, altered ceramide turnover, enrichment of ganglioside species, and coordinated remodeling of sphingolipid-related gene networks across glial populations. Together, these findings provide a spatial and cellular framework linking lipid metabolic resilience with protection against neurodegenerative pathology in the human brain, in a different way to healthy-like.

## METHODS

### Postmortem Human Samples

A total of 23 postmortem human brain samples were analyzed, including individuals with familial Alzheimer’s disease (FAD) carrying the PSEN1 E280A mutation, sporadic Alzheimer’s disease (SAD), and cognitively unimpaired controls. Two additional cases harboring the APOE-R154S (Christchurch) variant in one or both alleles were included for comparative analysis (Table 1). Neuropathological classification for all cases followed current Alzheimer’s disease diagnostic criteria (32).

Postmortem tissue and serum were obtained with informed consent and repository authorization, in accordance with the Belmont Report and with approval from the Institutional Review Board of the Universidad de Antioquia. Blood samples were collected in EDTA tubes, processed immediately, and stored at − 80°C until analysis. Brains were obtained within 12 h of death. One hemisphere was snapfrozen at − 80°C for biochemical analyses. For this study, dissections targeted the cingulate cortex, including adjacent portions of the corpus callosum and the subventricular zone. Detailed demographic, genetic, and neuropathological characteristics are provided in Table 1.

### Western Blotting

#### Western Blotting

Tissue samples comprising cingulate cortex together with the adjacent portion of the corpus callosum and the SVZ were homogenized in lysis buffer containing 150 mM NaCl, 20 mM Tris (pH 7.4), 10% glycerol, 1 mM EDTA, 1% NP-40 and protease inhibitors (1 mg/mL). Protein extracts (20 μg per lane) were denatured at 95°C for 3 min in loading buffer (0.375 M Tris, pH 6.8; 50% glycerol; 10% SDS; 0.5 M DTT; 0.002% bromophenol blue) and separated by SDS–PAGE using the Mini-PROTEAN system (Bio-Rad) with low–molecular weight standards. Proteins were transferred onto nitrocellulose (Bio-Rad 162 − 0115) or PVDF membranes (Bio-Rad 162–0177) using a Trans-Blot SD semi-dry transfer system (Bio-Rad) in Schafer–Nielsen buffer (48 mM Tris, 39 mM glycine, 20% methanol, pH 9.2) at 15 V for 50 min. Membranes were blocked in 5% nonfat dry milk prepared in TTBS (20 mM Tris–HCl, pH 7.5; 500 mM NaCl; 0.05% Tween-20) and incubated overnight at 4°C with primary antibodies (listed in Supplementary Table 1).

Fluorescent secondary antibodies were used for detection, and imaging was performed with the Odyssey Infrared Imaging System (LI-COR Biosciences, software v5.2). Densitometric analyses were carried out using the same software, and protein abundance was normalized to actin and expressed relative to control samples.

### Immunofluorescence

Free-floating tissue sections (30 μm) from the cingulate cortex, adjacent corpus callosum, and subventricular zone were obtained using a cryostat (Leica CM1850 UV). Sections were exposed to an LED light source for three consecutive nights to reduce autofluorescence, rinsed in 0.1 M phosphate buffer (PB), and subjected to antigen retrieval in citrate buffer (1×, pH 6.0; Master-Diagnostic MAD-004071R/D) at 95°C for 20 min. After additional PB washes, tissues were incubated in 50 mM ammonium chloride for 15 min to quench residual aldehydes and permeabilized with 0.3% Triton X-100. Sections were then washed and blocked for 1 h at room temperature in PB containing 0.3% Triton X-100 and 1% bovine serum albumin (BSA). Samples were incubated with primary antibodies for 72 h at 4°C, washed, and incubated with the corresponding secondary antibodies for 2 h at room temperature. Antibody sources and dilutions are listed in Supplementary Table 4. After final washes, sections were mounted using fluorescence-compatible medium. Imaging was performed using an Olympus IX81 epifluorescence microscope. Quantitative analyses were performed in ImageJ (version 1.54).

### Lipid Extraction and Analysis by Thin-Layer Chromatography (TLC)

All reagents, materials, and tissue homogenates were kept on ice or at 4°C throughout the procedure to preserve lipid integrity. Brain tissue from the cingulate cortex, adjacent corpus callosum, and subventricular zone was homogenized in Vance buffer (225 mM mannitol, 25 mM HEPES–KOH, pH 7.4, 1 mM EGTA, 2 mM MgCl_2_, 2 mM MnCl_2_) at a 10:1 buffer-to-tissue ratio. When required, homogenates were centrifuged to remove insoluble debris, and supernatants were used for downstream processing. Protein concentrations were determined using Bradford or BCA assays and normalized to 0.5–2 mg/mL.

Total lipids were extracted using a modified Folch procedure. Briefly, 2 mL of chloroform:ethanol (2:1, v/v) were added to each sample and vortexed. Subsequently, 1 mL of 0.9% NaCl and 1 mL of chloroform:methanol (2:1, v/v) were added sequentially with additional vortexing. Samples were centrifuged (3,500 rpm, 7 min, 4°C), and the lower organic phase was collected, dried under nitrogen or vacuum, and resuspended in chloroform to 15 mg/mL. For ganglioside-enriched fractions, the aqueous phase from the Folch extraction was subjected to two additional extractions with butanol to recover glycolipids, followed by ethanol washes to remove residual salts (33). Combined butanolic extracts were dried and resuspended in chloroform:methanol (2:1, v/v). For chromatographic separation, silica gel 60 aluminum-backed TLC plates (Merck) were prewashed with hexane and equilibrated in the appropriate mobile phase. Samples (15–20 μL at 15 mg/mL) were applied to each plate. Neutral lipids were resolved in hexane:diethyl ether:acetic acid (80:20:0.1, v/v/v); phospholipids in chloroform:methanol:acetic acid:water (50:30:8:4, v/v/v/v); sphingolipids in chloroform:methanol:0.25% KCl (60:30:8, v/v/v); and gangliosides in butanol:acetic acid:water (15.3:3.75:3.75, v/v/v). Plates were air-dried and visualized with anisaldehyde spray reagent. Chromatograms were digitized using a CAMAG TLC Scanner 3 (TLC Visualizer 3 | CAMAG), and absorbance was measured at 480 nm for semi-quantitative densitometric analysis, densitometric analysis was performed using visionCATS software (CAMAG).

### Sphingomyelinase Activity Assay and Thin-Layer Chromatography

All solutions, materials, and tissue extracts were maintained on ice or at 4°C and processed under minimal light exposure to preserve fluorescent products. Tissue from the cingulate cortex, adjacent corpus callosum, and subventricular zone was homogenized in Vance buffer (225 mM mannitol, 25 mM HEPES–KOH, pH 7.4, 1 mM EGTA, 2 mM MgCl_2_, 2 mM MnCl_2_) at a 10:1 buffer-to-tissue ratio. When required, homogenates were centrifuged to remove debris, and supernatants were collected. Protein concentration was determined by Bradford or BCA assays and adjusted to 0.5–2 mg/mL. For sphingomyelinase activity, 100 μL of protein extract (500 μg of protein) were incubated with 200 μL of reaction buffer (Vance buffer supplemented with 2 mM MgCl_2_, 2 mM MnCl_2_, and 1 μM BODIPY–sphingomyelin. Reactions were carried out at 37°C for 2 h in the dark. Lipids were extracted using a modified Folch procedure: 2 mL chloroform:ethanol (2:1, v/v) were added and vortexed, followed by the sequential addition of 1 mL 0.9% NaCl and 1 mL chloroform:methanol (2:1, v/v). Samples were centrifuged (3,500 rpm, 7 min, 4°C), and the organic phase was collected, dried under nitrogen or vacuum, and resuspended in chloroform to 15 mg/mL. TLC analyses were performed on silica gel 60 aluminum-backed plates (Merck), prewashed with hexane and equilibrated in the mobile phase. Samples were spotted alongside BODIPY–sphingomyelin and BODIPY–ceramide standards. Chromatographic separation was performed using chloroform:methanol:0.22% CaCl_2_ (60:35:8, v/v/v). After development, fluorescent lipids were visualized using a CAMAG TLC Scanner 3 (TLC Visualizer 3 | CAMAG), and BODIPY emission was recorded at 510 nm. Densitometric analysis was performed using visionCATS software (CAMAG). Sphingomyelinase activity was calculated as the ratio of BODIPY–ceramide product to remaining BODIPY–sphingomyelin substrate.

### Tissue Dissociation and Flow Cytometry Analysis

Cingulate cortex samples previously fixed in formaldehyde were washed three times with phosphate-buffered saline (PBS) for 5 min to remove residual fixative and then cut into small fragments (~ 1–2 mm^3^). Enzymatic dissociation was performed by incubating tissue fragments in PBS containing 0.1% collagenase type II (Sigma-Aldrich, C6885) and 0.01% DNase I (Sigma-Aldrich, DN25) at 37°C for 60 min, with gentle agitation every 10 min. Mechanical dissociation was achieved by gentle pipetting using a sterile 200 μL tip. The resulting suspension was passed through a 40–70 μm cell strainer (Falcon/Corning) to eliminate debris. Cells were centrifuged at 400 × g for 10 min, the pellet was resuspended in PBS, and this washing step was repeated twice to ensure removal of residual enzymes. Total cell number was quantified using a Neubauer hemocytometer before staining. For flow cytometry, cells were fixed and permeabilized in 0.1% Triton X-100 (Sigma-Aldrich, T8787) in PBS. Samples were incubated for 60 min at room temperature with the following primary antibodies (1:100 in PBS): Iba1 (Invitrogen, MA5-27726), GFAP (eBioscience, 14-9892-82), Ki-67 (Abcam, ab16667), and vimentin (Invitrogen, MA5-11883). After washing, cells were incubated with fluorophore-conjugated secondary antibodies (1:200 in PBS; Invitrogen) for 30 min at room temperature. Cell cycle assessment was performed by staining with propidium iodide (10–20 μg/mL; Sigma-Aldrich, P4170) for 15 min at room temperature. Samples were finally resuspended in PBS and analyzed on a BD LSR Fortessa flow cytometer (BD Biosciences), using identical acquisition settings for all samples. Human astrocytes and peripheral-blood macrophages fixed in 4% paraformaldehyde served as positive controls. Data analysis was conducted based on fluorescence intensity profiles to distinguish individual cellular populations.

### RNA Extraction and Digital PCR (dPCR) Quantification of DGAT2 and SMPD3 Expression

Absolute gene expression quantification was performed using digital PCR (dPCR) on the QuantStudio^™^ Absolute Q^™^ Digital PCR System (Thermo Fisher Scientific, USA). Total RNA was extracted from human cingulate cortex samples using the MagMAX^™^ mirVana^™^ Total RNA Isolation Kit (Thermo Fisher Scientific, A27828) following the manufacturer’s instructions. RNA purity and integrity were assessed spectrophotometrically, and concentrations were adjusted to 3.5 ng/μL for downstream dPCR reactions. One-step reverse transcription and amplification were performed using the Absolute Q^™^ 1-Step RT-dPCR Master Mix (4X) (Thermo Fisher Scientific, A55146). TaqMan^®^ Gene Expression Assays were used for DGAT2 (Assay ID Hs01045907_m1, VIC-labeled) and SMPD3 (Assay ID Hs00920354_m1, ABY-labeled). Reactions were loaded into Absolute Q^™^ Digital PCR chips and processed according to the manufacturer’s workflow for partition generation, thermal cycling, and fluorescence acquisition. Data were analyzed using the Absolute Q^™^ Analysis Suite v6.3.4, applying automated thresholding for partition-based quantification. Gene expression values were reported as 18S-normalized copy number values (target copies/μL relative to 18S rRNA copies/μL). All samples were run in technical duplicates, and non-template controls were included to exclude contamination or nonspecific amplification.

### Single-nucleus RNA Sequencing (snRNA-seq) Analysis

Publicly available human single-nucleus RNA sequencing datasets from Alzheimer’s disease and APOE genotype–defined cohorts were analyzed, including sporadic Alzheimer’s disease (SAD), familial Alzheimer’s disease (FAD), APOE3 Christchurch carriers, and non-demented controls (GEO accessions: GSE222494, GSE222495, GSE206744). Analyses were performed using a previously processed and annotated AnnData object containing postmortem cortical nuclei with metadata for diagnosis, sex, APOE genotype, sample identity, and major glial cell-type annotations. Downstream analyses were performed in Python using Scanpy and AnnData. Diagnostic labels were harmonized across datasets and grouped as Healthy, FAD, FAD APOE3Ch, and SAD. For the present study, analyses were focused on major glial populations, including astrocytes, microglia, mature oligodendrocytes, and oligodendrocyte precursor cells (OPCs). When required for visualization and gene-level comparisons, count matrices were library-size normalized, log-transformed, and scaled using standard Scanpy workflows. Dimensionality reduction and visualization were performed using PCA and UMAP embeddings, and glial populations were inspected using canonical lineage markers together with the available curated cell-type annotations. To evaluate lipid-associated transcriptional remodeling, we curated a candidate gene panel related to the lipid classes analyzed experimentally in postmortem tissue, including genes involved in sterol and cholesterol handling, triacylglycerol synthesis and lipid-droplet biology, phospholipid remodeling, fatty-acid activation/transport, and sphingolipid metabolism. Candidate genes were selected from the detected transcriptome based on functional relevance to the biochemical lipid pathways evaluated in the study and then organized into lipid-class modules for downstream visualization. Gene-expression patterns were summarized across diagnostic groups and glial populations using heatmaps, dot plots, and average-expression matrices. Vimentin-associated glial remodeling was evaluated by mapping VIM expression across the glial UMAP and by quantifying VIM-positive or VIM-enriched nuclei across glial populations and diagnostic groups. Astrocytes, microglia, mature oligodendrocytes, and OPCs were subsequently analyzed separately to determine whether lipid-metabolism signatures were broadly shared across glia or preferentially associated with specific cell populations. For differential expression analyses, a pseudo-bulk strategy was used to reduce cell-level dependency and account for inter-individual variability. Briefly, raw or count-like expression values were aggregated at the sample-by-cell-type level, retaining sample–cell-type combinations with sufficient nuclei for robust analysis. Pseudo-bulk differential expression was then performed using pyDESeq2, comparing FAD, FAD APOE3Ch, and SAD groups against Healthy controls, as well as FAD APOE3Ch against FAD where appropriate. Differentially expressed genes were prioritized using nominal statistical significance and effect-size directionality, with particular emphasis on genes belonging to the curated lipid-metabolism modules. To explore biological programs associated with glial lipid remodeling, differentially expressed genes from each glial population and diagnostic contrast were analyzed for functional enrichment using Gene Ontology and curated pathway collections through Python-based enrichment workflows. Enrichment results were summarized by glial population and diagnostic group to identify convergent and cell-type-specific pathways associated with AD and APOE3Ch-related glial states. Cell-cycle heterogeneity was evaluated using canonical phase-associated gene sets and Scanpy’s cell-cycle scoring functions. Cell-cycle scores and metadata variables, including sex, diagnosis, APOE genotype, sample identity, and glial annotation, were retained for downstream stratification and interpretation. All analyses were performed using Python-based single-cell workflows, including Scanpy, AnnData, pandas, NumPy, matplotlib/seaborn, gseapy, and pyDESeq2.

### Protein–protein interaction network analysis

Protein–protein associations were obtained from the STRING database (accessed 23 November 2025; https://string-db.org). We imported the list of differentially represented proteins (gene names / UniProt IDs) and generated organism-specific networks, including both physical and functional associations, using the “full STRING network” view. Interactions were filtered at a minimum combined confidence score ≥ 0.70 (high confidence) and displayed edge weights corresponding to STRING’s combined score (which integrates experimental data, curated databases, text mining, and other evidence channels). Networks were exported in tabular form (edge list and node attributes) via STRING’s “Tables/Exports” function for downstream analysis and visualization. Clustering of network modules was performed with the Markov Cluster Algorithm (MCL) implemented in STRING/Cytoscape (inflation = 2.0) to identify highly interconnected protein groups. Functional enrichment (Gene Ontology biological process, KEGG pathways, Pfam/InterPro domains, and UniProt keywords) was performed using STRING’s built-in over-representation test (hypergeometric test) and p-values were adjusted for multiple testing by the Benjamini–Hochberg procedure; terms with FDR ≤ 0.05 were considered significant. For programmatic reproducibility, we confirmed results using the STRING API / STRINGdb package and archived the exact query outputs (edge lists, enrichment tables, and parameters) as source data.

## RESULTS

### APOE3Ch increases proteins associated to lipid synthesis and lipid symporter receptor

**Figure 1:**
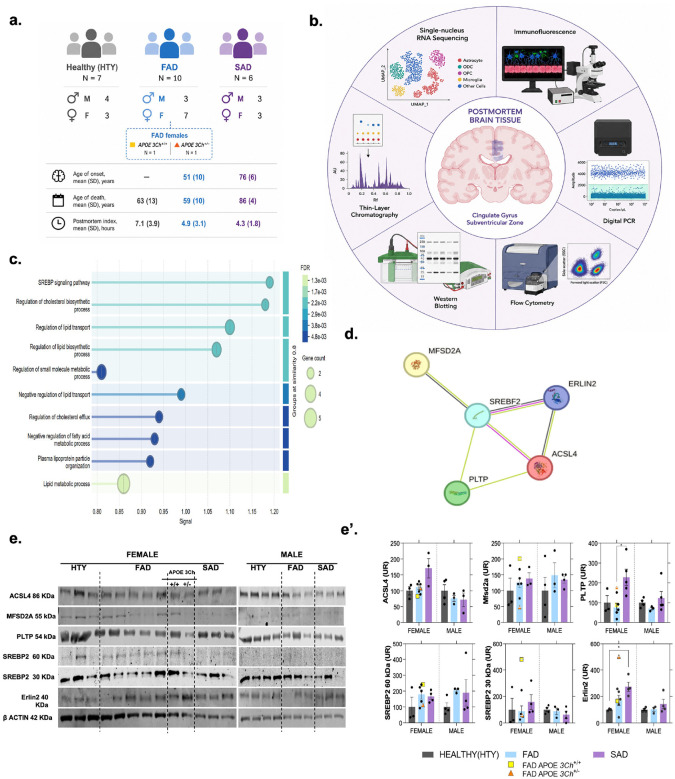
Postmortem cohort and lipid metabolism–related protein expression. **(a)** Summary of the human postmortem cohort, including healthy controls (HTY), familial Alzheimer’s disease cases carrying the PSEN1 E280A mutation (FAD), FAD APOE3Ch carriers, and sporadic Alzheimer’s disease cases (SAD), with sex distribution, age of onset, age of death, and postmortem index **(b)** Schematic representation of postmortem tissue sampling (cingulate, SVZ and corpus callosum) and analytical approaches including immunofluorescence, dPCR, flow cytometry, Western blotting, thin layer chromatography, snRNA-seq (created with BioRender.com) **(c)** Biological process enrichment analysis of the selected lipid-related protein panel, showing enriched functional terms, FDR values, and gene counts. **(d)** Protein interaction network showing functional connectivity among the analyzed lipid metabolic targets, including ACSL4, MFSD2A, PLTP, SREBP2/SREBF2, and Erlin2. **(e)** Western blots and **(e’)** corresponding quantifications of proteins involved in lipid regulation (Erlin2, SREBP2), transport (MFSD2A, PLTP), and fatty acid activation (ACSL4). Data are expressed as mean ± SEM; significance: *p < 0.05, **p < 0.01, ***p < 0.001 (N = 23). For women, groups include healthy controls (HTY, n = 3), FAD–PSEN1 E280A carriers (n = 5), FAD APOE3Ch+/+ carriers (n = 1), FAD APOE3Ch+/− heterozygous carriers (n = 1), and SAD cases (SAD, n = 3). For men, groups include HTY (n = 4), FAD–PSEN1 E280A carriers (n = 3), and SAD (n = 3). Parametric comparisons were performed using one-way ANOVA, whereas non-parametric data were analyzed using the Kruskal–Walli’s test.

Guided by systemic lipid alterations in familial and sporadic AD, which highlighted the general reduction of lipid in serum from APOE-3Ch (31), we investigated whether lipid metabolic remodeling extends to vulnerable brain regions. We assembled a postmortem cohort comprising controls, FAD, FAD APOE3Ch carriers, and SAD cases (Fig. 1a), and profiled the cingulate cortex and adjacent corpus callosum–SVZ axis using complementary biochemical, spatial, cytometric and transcriptomic (Fig. 1b). A targeted protein panel selected to cover fatty acid activation, lipid transport, cholesterol regulation, and ER-associated lipid homeostasis identified a coherent lipid metabolic module involving ACSL4, MFSD2A, PLTP, SREBP2/SREBF2, and Erlin2, with functional annotation confirming their association with lipid transport, cholesterol biosynthesis, cholesterol efflux, lipoprotein particle organization, and SREBP signaling (Fig. 1c). The protein interaction network further showed functional connectivity among these proteins, with SREBP2/SREBF2 occupying a central position within the module (Fig. 1d). This module was remodeled in a sex- and diagnosis-dependent manner (Fig. 1e, e’). SAD females showed increased ACSL4, PLTP, and Erlin2, whereas FAD APOE3Ch females displayed higher SREBP2 and MFSD2A. These findings indicate that AD-associated lipid alterations are detectable within the cingulate cortex–white matter/SVZ axis and suggest that APOE3Ch resilience is accompanied by a distinct reorganization of sterol-regulatory and lipid transport programs rather than preservation of a control-like state.

#### Generalized reduction of neutral lipids in AD and divergent TAG storage in FAD-APOE-3Ch in the cingulate cortex

**Figure 2:**
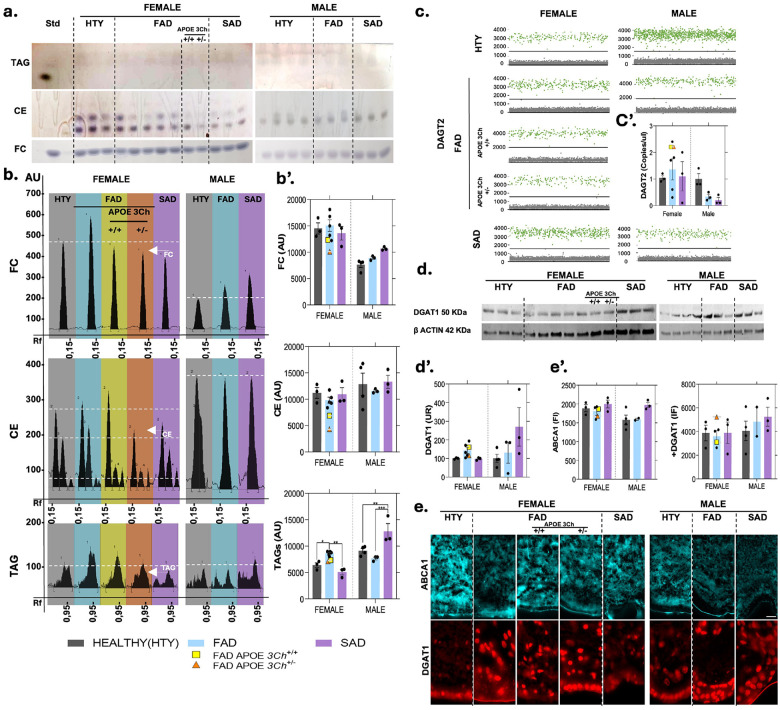
Analysis of neutral lipid metabolism in postmortem cingulate. **(a)**Representative thin-layer chromatography (TLC) plate showing the separation of neutral lipid classes, including free cholesterol (FC), cholesteryl esters (CE), and triacylglycerols (TAG). **(b–b’)** Representative TLC scan and corresponding quantifications of neutral lipid classes. **(c)**Representative scatter plots of individual dPCR reactions acquired using the ThermoFisher Q Studio dPCR platform, showing detection of DGAT2 mRNA in post-mortem cingulate tissue from women (left panel) and men (right panel). Each green dot corresponds to a positive droplet, defined by fluorescence above the automated threshold, whereas grey dots represent negative droplets. DGAT2 expression values were normalized to 18S rRNA. **(d)** Western blots and **(d’)** Western blot quantification of DGAT1 protein levels. **(c’)** Bar plot showing the absolute quantification of DGAT2 (copies/μL) across diagnostic groups. **(e’)** Quantification of immunofluorescence (IF) signal in the SVZ of the cingulate cortex, showing localization patterns of DGAT1 and ABCA1. Data are expressed as mean ± SEM; significance: *p < 0.05, **p < 0.01, ***p < 0.001 (N = 23). For women, groups include healthy controls (HTY, n = 3), FAD–PSEN1 E280A carriers (n = 5), carriers with the protective FAD APOE3Ch+/+ variant (n = 1), FAD APOE3Ch+/− heterozygous carriers (n = 1), and sporadic AD cases (SAD, n = 3). For men, groups include HTY (n = 4), FAD–PSEN1 E280A (n = 3), and SAD (n = 3). Parametric comparisons were performed using one-way ANOVA, whereas non-parametric data were analyzed using the Kruskal–Walli’s test.”

Next, we profiled neutral lipid including free cholesterol (FC), cholesteryl esters (CE), and triacylglycerols (TAG), in the cingulate cortex (Fig. 2a). TLC-based lipid profiling showed that cholesterol-associated lipid pools were comparatively preserved at the group level, with FC and CE showing no significant overall differences across diagnostic categories (Fig. 2b, b’). However, sex-stratified analysis revealed disease-associated trends: females with FAD showed a modest increase in FC and a decrease in CE, whereas males did show variation in both lipid classes. Notably, female APOE3Ch carriers showed lower FC and CE levels relative to the broader FAD group, suggesting that APOE3Ch-associated remodeling may involve altered cholesterol synthesis rather than accumulation of cholesterol-related lipid pools. In contrast to the relatively subtle changes in FC and CE, TAG showed a significant disease- and sex-dependent pattern. TAG levels were increased in FAD females compared with healthy controls and SAD cases, with APOE3Ch carriers broadly following the FAD-associated TAG profile (Fig. 2b, b’). In males, TAG accumulation was higher in SAD. These data indicate that neutral lipid dysregulation remodeling is not uniformly coupled in AD cingulate cortex.

To further contextualize these neutral lipid changes, we examined molecular regulators related to TAG synthesis and cholesterol handling. Because DGAT1 and DGAT2 catalyze the terminal step of TAG synthesis and represent distinct components of the TAG biosynthetic machinery (33), we evaluated DGAT2 transcript levels by dPCR and DGAT1 protein abundance by Western blot as complementary readouts of TAG regulation (Fig. 2c, c’). DGAT2 was markedly reduced in FAD and SAD males without changes in females; DGAT1 protein levels more closely paralleled the TAG profile, supporting an association between DGAT1 and cortical TAG accumulation in the FAD cases (Fig. 2d, d’). Complementarily, tissue immunofluorescences for DAGT1 and ABCA1 did not present changes in the intensity, but DGAT1 IF in FAD females independent of APOE isoform revealed a loss of cell stratification SVZ (Fig. 2e–e’).

### Phospholipid depletion in FAD and SAD cases, but with reduced cPLA2 activation in APOE3Ch

**Figure 3:**
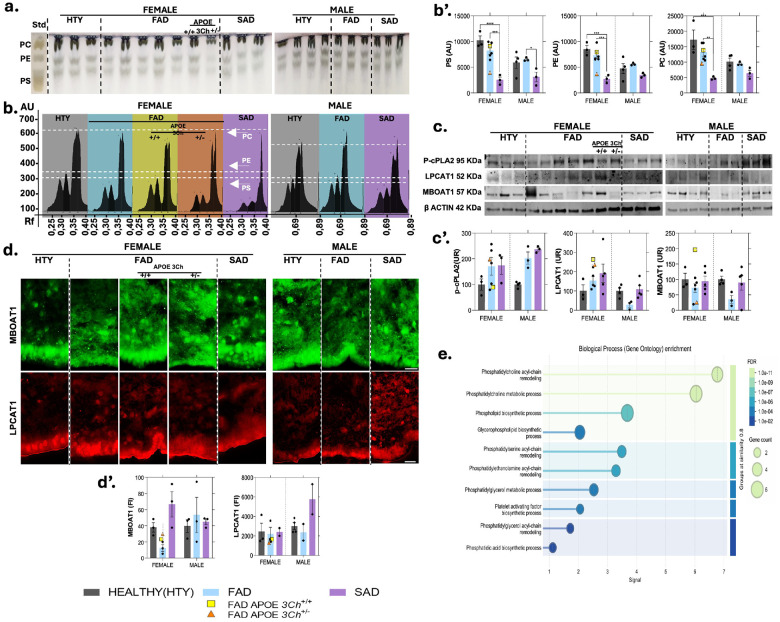
Analysis of phospholipid metabolism and remodeling enzymes in postmortem cingulate. **(a)**Representative thin-layer chromatography (TLC) plate showing the separation of major phospholipid classes, including phosphatidylserine (PS), phosphatidylethanolamine (PE), and phosphatidylcholine (PC) **(b–b’)** Representative TLC scan and corresponding quantification of phospholipid classes. **(c)** Western blots and **(c’)** Western blot quantification of phospholipid remodeling enzymes, including cPLA_2_, LPCAT1, and MBOAT1. **(d-d’)** immunofluorescence (IF) signal in cortical tissue, depicting MBOAT1 and LPCAT1 colocalization patterns and their Quantification. Data are expressed as mean ± SEM; significance: *p < 0.05, **p < 0.01, ***p < 0.001 (N = 23). For women, groups include healthy controls (HTY, n = 3), FAD–PSEN1 E280A carriers (n = 5), FAD APOE3Ch+/+ carriers (n = 1), FAD APOE3Ch+/− heterozygous carriers (n = 1), and sporadic AD cases (SAD, n = 3). For men, groups include HTY (n = 4), FAD–PSEN1 E280A carriers (n = 3), and SAD (n = 3). Parametric comparisons were performed using one-way ANOVA, whereas non-parametric data were analyzed using the Kruskal–Walli’s test. **(e)** Gene Ontology (GO) enrichment analysis of biological processes associated with the analyzed proteins.

Next, we evaluated how the membrane-forming phospholipids were remodeled in the AD cingulate cortex. TLC-based profiling of phosphatidylserine (PS), phosphatidylethanolamine (PE), and phosphatidylcholine (PC) revealed a coordinated depletion of major structural phospholipid classes in both FAD and SAD females, with a most pronounced reductions in SAD (Fig. 3a, b, b’). This pattern was also similar in males with SAD. Protein analysis revealed increased p-cPLA2 in both FAD and SAD, consistent with engagement of phospholipid hydrolysis/remodeling pathways in AD cortex (Fig. 3d, d’). Although LPCAT1 and MBOAT1 enzymes did not change. Interestingly, APOE3Ch homozygous presented a reduction of p-cPLA2 and increase at the acyltranferases (Fig. 3c, c’), whereas the heterozygous case showed higher p-cPLA2 and lower MBOAT1 relative to the homozygous carrier. This pattern suggests that APOE3Ch-associated remodeling may involve a shifted hydrolysis-reacylation balance, rather than preservation of baseline phospholipid composition (Fig. 3c, c’). In the tissue, LPCAT1 IF presented a differential pattern of layering at the layer I and II of the SVZ in all FAD females (Fig. 3d, d’) and MBOAT1 cells were more disperse in SAD conditions. These findings identify a membrane phospholipid remodeling (Fig. 3e), contextualizing that APOE3Ch-associated resilience may involve remodeling of membrane lipid homeostasis rather than complete protection of phospholipid loss.

### Ganglioside turnover by APOECh in the cingulate cortex

**Figure 4:**
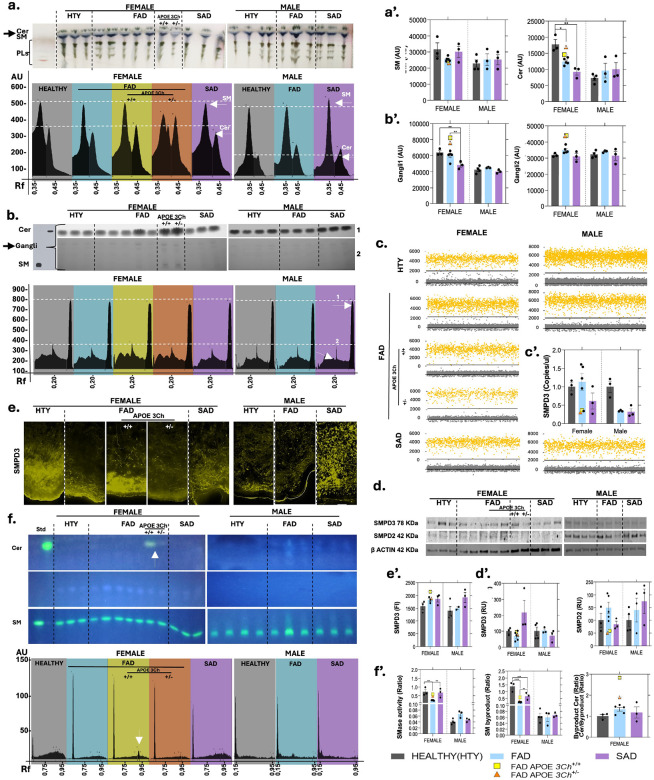
Sphingolipid metabolism and enzymatic regulation in postmortem cingulate cortex. **(a-b)**Representative thin-layer chromatography (TLC) plate and scan showing the separation of major sphingolipid classes, including sphingomyelin (SM), ceramide (Cer) **(a)**, and ganglioside-enriched fractions **(b)**. **(a’–b’)**Corresponding quantifications from TLC. **(c)** Representative scatter plots of individual dPCR reactions acquired using the ThermoFisher Q Studio dPCR platform, showing detection of SMPD2 mRNA in post-mortem cingulate tissue from women (left panel) and men (right panel). Each yellow dot corresponds to a positive droplet, defined by fluorescence above the automated threshold, whereas grey dots represent negative droplets. SMPD2 expression values were normalized to 18S rRNA. **(c’)** Bar plot showing the absolute quantification of SMPD3 (copies/μL) across diagnostic groups. Quantification of *smpd3* expression by digital dPCR. **(d–d’)** Quantification of SMPD2 and SMPD3 protein levels by western blot. **(e–e’)** Representative immunofluorescence (IF) images showing the cortical distribution of SMPD3 and corresponding fluorescence quantifications. **(f)** Representative enzymatic activity assay of sphingomyelinase (SMase) a scan **(f’)** and corresponding quantifications. Data are expressed as mean ± SEM; significance: *p < 0.05, **p < 0.01, ***p < 0.001 (N=23). For women, groups include healthy controls (HTY, n=), FAD–PSEN1 E280A carriers (n=5), carriers with the protective APOE3Ch+/+ variant (n=1), APOE3Ch+/− heterozygous carriers (n=1), and sporadic AD cases (SAD, n=3). For men, groups include HTY (n = 4), FAD–PSEN1 E280A (n=3), and SAD (n=3). Parametric comparisons were performed using one-way ANOVA, whereas non-parametric data were analyzed using the Kruskal–Walli’s test.”

Sphingolipids, a major class of membrane lipids involved in cellular signaling, glial biology, and stress-associated responses. TLC-based profiling revealed a disease-associated reduction in ceramide (Cer) levels in female FAD and SAD cortices relative to healthy controls, with the most pronounced decrease observed in SAD (Fig. 4a, a’). In contrast, APOE3Ch carriers maintained relatively higher Cer levels compared with the broader FAD group. We then evaluated ganglioside-enriched fractions as readouts of complex glycosphingolipid-associated membrane remodeling. Both the less polar ganglioside-enriched fraction and the more polar fraction were increased in APOE3Ch carriers (Fig. 4b, b’). Together with the relative preservation of Cer, these findings indicate that APOE3Ch carriers exhibit a sphingolipid profile that differs from both FAD and SAD cortices.

To determine whether these lipid class changes were accompanied by alterations in sphingomyelinase-related machinery, we analyzed SMPD transcripts and proteins. SMPD2 transcript levels showed no consistent group-level differences by dPCR (Fig. 4c, c’), indicating that transcriptional variation alone did not explain the sphingolipid phenotype. At the protein level, however, APOE3Ch carriers showed a distinct pattern characterized by increased SMPD3 and reduced SMPD2 (Fig. 4d, d’). This protein-level divergence supports reorganization of sphingolipid regulatory machinery in APOE3Ch-associated AD brain tissue. SMPD3 IF confirmed an enhanced SMPD3 signal in FAD APOE3Ch with enrichment in a layer II of SVZ. (Fig. 4e, e’). This finding extends the laminar changes observed for neutral lipid- and phospholipid-associated proteins (Fig. 2, 3), indicating that sphingolipid remodeling is also coupled to altered neuroanatomical niche. Complementarily, sphingomyelinase (SMase) activity was elevated in FAD APOE3Ch, aligning with preserved Cer pools and enhanced turnover (Fig. 4f–f’, arrow).

### Glial vimentin + cells present a distinguishing feature in FAD APOE-3Ch phenotype in SVZ cingulate cortex

**Figure 5:**
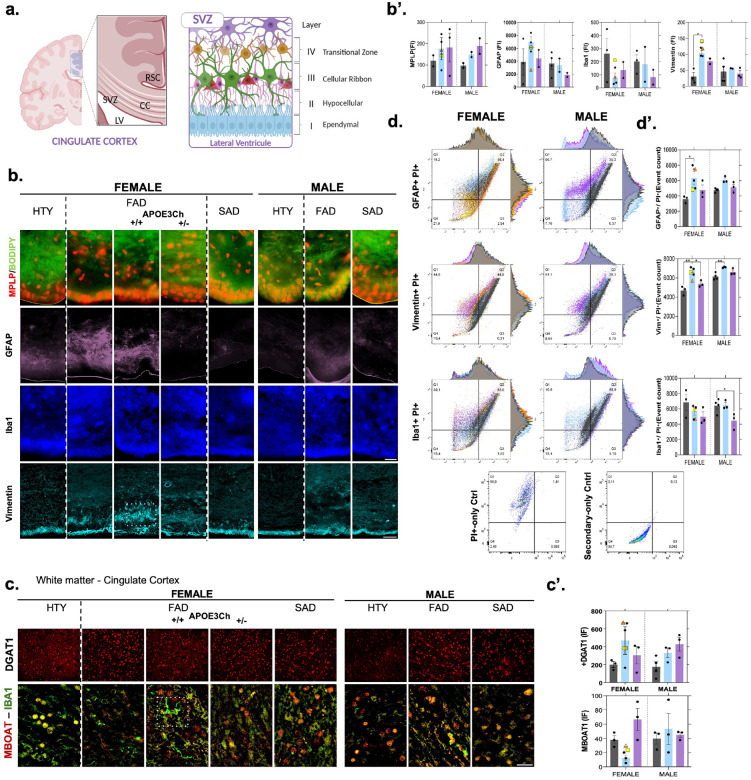
Glial remodeling in postmortem cingulate cortex and adjacent regions. **(a)**Representative schematic of the cingulate cortex, corpus callosum, and subventricular zone (SVZ), showing their anatomical location (left) and laminar organization (right). Layers are defined as: I, ependymal; II, hypocellular; III, cellular ribbon; IV, transition zone. **(b-b’)** Representative immunofluorescence (IF) images showing MPLP (red), BODIPY (green), GFAP (magenta), IBA1 (blue), and Vimentin (cyan) labeling across groups and genotypes, used for glial characterization of the SVZ, together with the corresponding fluorescence quantifications. MPLP, BODIPY, GFAP, and IBA1 panels were acquired at 60X magnification (scale bar=20 μm). Vimentin and DGAT1 at 20X magnification (scale bar=50 μm). **(c–c’)** IF staining of DGAT1 (red), MBOAT1 (red), and IBA1 (green) in the cingulate cortex and corpus callosum, illustrating molecular and cellular alterations across conditions. **(d–d’)** Flow cytometry analysis and corresponding quantifications of GFAP^+^, Vimentin^+^, and IBA1^+^ populations. Data are expressed as mean ± SEM; significance: *p < 0.05, **p < 0.01, ***p < 0.001 (N=23). For women, groups include healthy controls (HTY, n= 3), FAD–PSEN1 E280A carriers (n=5), carriers with the protective FAD APOE3Ch+/+ variant (n=1), FAD APOE3Ch+/− heterozygous carriers (n=1), and sporadic AD cases (SAD, n=2–3). For men, groups include HTY (n = 2–4), FAD–PSEN1 E280A (n=2–3), and SAD (n=2–3). Parametric comparisons were performed using one-way ANOVA, whereas non-parametric data were analyzed using the Kruskal–Walli’s test”

Next, we analyze and focus on main changes observed in white matter (WM) and SVZ of postmortem brains from familial FAD, SAD, compared with healthy controls (HTY). Immunofluorescence revealed an increased redistribution of glial cells, mainly Oligodendrocytes (MPLP+), Astrocytes (Vimentin + and GFAP+) at layers I and II of the SVZ in FAD, especially in females and a particular reduction of microglia (Iba1) in SAD (Fig. 5a, b, b’). Also, we found more DGAT1 positive cells in white matter of female FAD (Fig. 5c, c’). Changes on cell morphology of Vimentin + and Iba1 + cells were observed under APOE3Ch homozygous with a more expanded phenotype for astrocytes and microglia, respectively (Fig. 5b). Flow cytometry confirmed an increase in GFAP^+^ and Vimentin^+^ populations, predominantly in FAD, accompanied by a reduction in Iba1^+^ cells in SAD (Fig. 5d, d’). Importantly, cell-cycle analysis by propidium iodide (PI) staining revealed increased cell-cycle activity across all phases (G1, S, and G2/M), with the most prominent shifts occurring in Vimentin^+^ populations, particularly in females with FAD (Supp. Figure 1).

### Glial sphingolipids metabolism genes involved in FAD APOE-3Ch protection in prefrontal cortex

**Figure 6:**
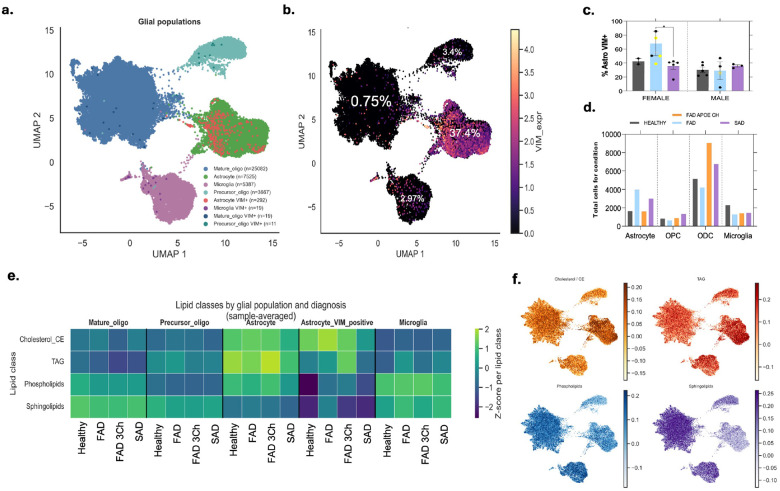
Single-nucleus transcriptomic profiling and lipid metabolism signatures in Alzheimer’s disease. **(a)** UMAP visualization of glial nuclei grouped into four major populations: astrocytes, microglia, mature oligodendrocytes (ODC), and oligodendrocyte precursor cells (OPC). **(b)**UMAP feature plot showing the distribution of VIM expression across glial populations and the relative proportion of VIM-positive nuclei. **(c)**Quantification of the percentage of VIM-positive astrocytes across diagnostic groups and sex. (**d**) Distribution of glial cell types according to Alzheimer’s disease diagnosis. Data are shown as mean ± SEM. Statistical comparisons were performed using ANOVA; ***p < 0.001. **(e)** Heatmap showing the expression of genes associated with major lipid classes across glial populations, including genes related to sterol/cholesterol metabolism, triacylglycerol handling, phospholipid remodeling, fatty-acid metabolism, and sphingolipid pathways. (**f**) UMAP feature plot showing the distribution of lipid clases across glial populations. Single-nucleus RNA-seq analyses were performed using samples from HTY controls (n = 3), FAD–PSEN1 E280A carriers (n = 3), FAD APOE3Ch+/+ carriers (n = 1), FAD APOE3Ch+/− carriers (n = 2), and SAD cases (n = 3), for a total of N = 12 individuals. Unless otherwise indicated, analyses were performed without sex stratification.

The spatial and cytometry analyses identified Vimentin-positive glial remodeling as a prominent cellular feature of the cingulate-SVZ axis. To define the molecular programs associated with this phenotype, we analyzed single-nucleus RNA-seq data from postmortem prefrontal cortical region (GNA’s bank available in GEO accessions: GSE222494, GSE222495, GSE206744). We focused on major glial populations, including astrocytes, microglia, mature oligodendrocytes, and oligodendrocyte precursor cells. UMAP visualization resolved these glial compartments, with mature oligodendrocytes representing the most abundant population, followed by astrocytes, oligodendrocyte precursor, and microglia (Fig. 6a). We next examined the distribution of VIM expression across glial lineages. VIM expression was not uniformly distributed, but was preferentially enriched in astrocytes, where VIM-positive nuclei represented 37.4% of the astrocytic compartment, whereas lower proportions were detected in microglia, oligodendrocyte precursor, and mature oligodendrocytes (Fig. 6b). Because sex-stratified analyses retained biologically relevant information most clearly for this readout, we specifically evaluated the proportion of VIM-positive astrocytes by sex and diagnosis. In females, both FAD and FAD APOE3Ch cases showed increased VIM-positive astrocyte proportions relative to healthy controls and SAD cases (Fig. 6c). Being more abundant ODC in all AD conditions, mainly in FAD-APOE3Ch and astrocytes in FAD cases (Fig. 6d).

To determine whether this glial remodeling was linked to lipid-associated transcriptional programs, we analyzed a curated set of genes related to the lipid classes examined throughout the study, including sterol metabolism, TAG/glycerolipid metabolism, phospholipid remodeling, and sphingolipid metabolism. This targeted analysis revealed that lipid-associated gene expression was organized by both glial identity and diagnosis (Fig. 6e). Sterol-associated genes were increased mainly in astrocytes and VIM-positive astrocytes. TAG-associated genes were also higher in astrocytic populations, with the strongest increase in VIM-positive astrocytes from FAD APOE3Ch cases. Phospholipid-related genes showed a broader but more moderate increase across mature oligodendrocytes, astrocytes, and microglia. In contrast, sphingolipid-associated genes were most prominently increased in mature oligodendrocytes, with additional signal in oligodendrocyte precursor and microglia (Fig. 6f).

**Figure 7:**
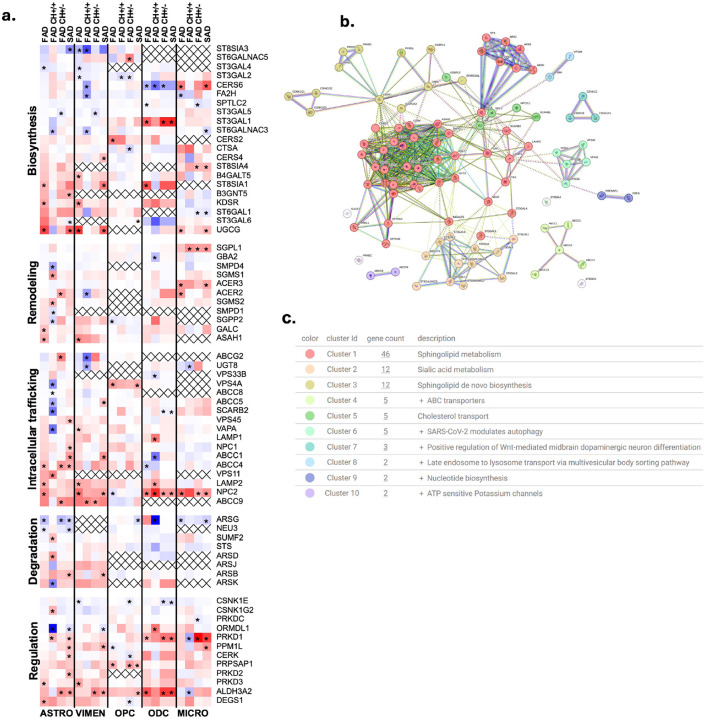
Single-nucleus transcriptomic profiling of sphingolipid metabolism in glial populations. **(a)**Heat map fold change analysis of differentially expressed genes from single-cell data (blue, negative; white, no change; red, positive); statistical significance indicated by asterisks (*p < 0.05). **(b)** Clustering of genes based on co-expression patterns. **(c)** Functional annotation of gene clusters (N = 12). In this analysis, samples were not stratified by sex and included healthy controls (HTY, n = 3), FAD–PSEN1 E280A carriers (n = 3), APOE3Ch+/+ carriers (n = 1), APOE3Ch+/− carriers (n = 2), and sporadic AD cases (SAD, n = 3).

Consistent with this cell type–specific sphingolipid signal, we further resolved sphingolipid-related genes into biosynthesis, remodeling, intracellular trafficking, degradation, and regulatory categories. The heatmap showed that these programs were not uniformly altered across glial lineages but followed diagnosis- and cell type–dependent patterns, with APOE3Ch-associated cases showing a prominent reduction in degradation-related genes and selected immune/regulatory genes (Fig. 7a). STRING-based interaction mapping and cluster annotation further organized these genes into connected modules related to sphingolipid metabolism, sialic acid metabolism, *de novo* sphingolipid biosynthesis, ABC transporters, cholesterol transport, and autophagy-associated processes (Fig. 7b, c).

## Discussion

The present study provides an integrative analysis of lipid metabolic and glial remodeling in postmortem cerebral cortex from individuals with familial and sporadic Alzheimer’s disease, including rare carriers of the APOE3 Christchurch variant. We identify a distinctive metabolic signature associated with APOE3Ch, characterized by sterol-regulatory and lipid transport changes, a shifted phospholipid hydrolysis–reacylation balance, and a sphingolipid metabolic state compatible with enhanced lipid turnover and recycling. Rather than indicating preservation of a healthy-like lipid state, these findings suggest that APOE3Ch-associated resilience may involve an alternative lipid–glial remodeling program across the cingulate cortex–corpus callosum/SVZ axis, potentially attenuating lipid-driven neuroinflammatory responses and reduced pruning of oligodendrocytes.

A prominent finding in our neutral lipid profiling is the clear dissociation between cholesterol-associated lipid pools and triacylglycerol (TAG) storage responses within the AD cortex. While free cholesterol (FC) and cholesteryl esters (CE) remained relatively stable at the group level, cortical TAG levels were significantly elevated in familial AD (FAD) females. This accumulation was paralleled by increased DGAT1 protein abundance, an association between DGAT1-linked TAG synthesis and cortical neutral lipid storage under AD-related stress, consistent with recent descriptions of lipid-droplet accumulation under neuroinflammatory or dysfunctional glial conditions (35). In parallel, increased ACSL4, PLTP, Erlin2, and p-cPLA2 in SAD females, and to a lesser extent in FAD females, suggests engagement of fatty acid activation, lipid transfer, ER-associated lipid regulation, and phospholipid remodeling pathways (36, 37).

Within this landscape, female *APOE3Ch* carriers showed lower FC and CE levels while executing a distinct homeostatic feedback look via the robust upregulation of the master sterol regulator SREBP2 and the lipid symporter transporter MFSD2A. This implies that *APOE3Ch*-mediated tissue resilience does not depend on a global prevention of storage lipid pathways, but may involve compensatory reorganization of sterol sensing, lipid uptake, and lipid transport programs. This interpretation is consistent with the central position of SREBP2/SREBF2 in the protein interaction network and with the broader role of APOE-dependent lipid transport in AD-related vulnerability (38).

Beyond storage responses, our data show coordinated depletion of major membrane-forming phospholipid classes in AD. We observed a coordinated depletion of major structural phospholipids, including phosphatidylserine (PS), phosphatidylethanolamine (PE), and phosphatidylcholine (PC), across both FAD and sporadic AD (SAD) cases. This broad depletion was functionally mirrored by the robust upregulation of phosphorylated cytosolic phospholipase A2 (p-cPLA2), consistent with increased engagement of phospholipid hydrolysis and remodeling pathways (39). Strikingly, *APOE3Ch* carriers did not display baseline preservation of structural phospholipids. Instead, the homozygous carrier presented a shifted hydrolysis–reacylation profile, characterized by lower p-cPLA2 expression alongside elevated lysophospholipid acyltransferases-related proteins. This pattern suggests that APOE3Ch-associated resilience may involve active membrane lipid remodeling rather than passive protection from phospholipid loss (40, 41). Such remodeling may help preserve acyl-chain homeostasis, including fatty acid fractions relevant for synaptic and membrane function, such as docosahexaenoic acid (DHA) (42).

Sphingolipid analyses provide further compelling evidence of a distinct *APOE3Ch*-associated tissue profile. While standard FAD and SAD cortices exhibited a marked reduction in total ceramide (Cer) levels, *APOE3Ch* carriers maintained significantly higher ceramide pools coupled with a profound enrichment in both polar and less-polar ganglioside-containing fractions. Enzymatically, this metabolic signature was driven by a striking protein-level divergence, an elevation of neutral sphingomyelinase 3 (SMPD3) and a suppression of acid sphingomyelinase (SMPD2), which synchronized with elevated total sphingomyelinase (SMase) catalytic activity. Because ceramide acts as a central hub in the sphingolipid metabolism, its selective preservation, allied to increased complex gangliosides-enriched fractions, suggests altered sphingomyelinase-dependent turnover and enhanced sphingolipid recycling/salvage activity (43). Rather than exhausting structural lipids or generating pro-apoptotic signals, the *APOE3Ch* configuration may favor redistribution of sphingolipid metabolism toward complex glycosphingolipid-associated membrane states. These ganglioside-enriched membrane microenvironments could drastically modulate how neurotoxic amyloid-β (Aβ) species are partitioned, embedded, or cleared within the cortical tissue (44).

Crucially, these metabolic reconfigurations are not uniform tissue-wide phenomena but are tightly coupled to cellular and spatial remodeling across the cingulate cortex–corpus callosum/SVZ axis. Immunofluorescence revealed a distinct spatial redistribution of lipid processing machinery (such as ABCA1, DGAT1, LPCAT1, and SMPD3) across the SVZ layers. These findings suggest that lipid remodeling in AD is closely linked to glial reorganization of the ventricular wall and white matter-associated niche, rather than being restricted to bulk cortical lipid changes (45). Single-nucleus transcriptomic profiling revealed significant remodeling of lipid metabolic gene expression across glial populations in prefrontal cortex, highlighted Vimentin-positive astroglial states, mainly associated to sphingolipids and gangliosides recycling; even sialylation in oligodendrocytes and sialylation, maybe represented in an increased differentiated oligodendrocytes (46) and reduced immune response.

We acknowledge that this study has specific limitations. Due to the rarity of human *APOE3Ch* postmortem tissue, our carrier cohort is necessarily limited in sample size, meaning these discoveries must be interpreted as hypothesis-generating. Additionally, while thin-layer chromatography offers highly robust, quantitative data on major lipid classes and fractions, it does not possess the high-resolution capacity to differentiate specific fatty-acyl chain configurations or saturation levels within individual ceramide or ganglioside species. Moreover, the single-nucleus RNA-seq data were obtained from postmortem prefrontal cortical regions, whereas spatial and biochemical validation focused on the cingulate cortex–corpus callosum/SVZ axis; therefore, these datasets should be interpreted as complementary but not anatomically identical. Finally, SVZ-adjacent laminar patterns were inferred from tissue organization and imaging context but were not independently delineated using layer-specific anatomical markers.

In conclusion, our results demonstrate that *APOE3Ch*-associated clinical resilience is characterized by a distinct lipid–glial remodeling state rather than preservation of a healthy baseline profile. By executing a distinct sterol, phospholipid, and sphingolipid-salvage program linked directly to spatial astroglial reorganization across the cingulate-SVZ axis, the *APOE3Ch* brain displays an optimized tissue configuration to tolerate advanced neurodegenerative pathology. These findings illuminate the critical role of lipid-glial remodeling as a major axis of AD heterogeneity and highlight specific sphingolipid and membrane-remodeling pathways as highly viable mechanisms for future studies of resilience in Alzheimer’s disease.

## Supplementary Files

This is a list of supplementary files associated with this preprint. Click to download.

- Table1Clinicalcharacteristicofpostmortemcases..pptx
- SuppTable1ComprehensiveReagentandAntibodyInventoryforExperimentalReproducibility.xlsx
- SupplFig1CellcycleprofilesmeasuredbyPIsignal.pptx

Tables are available in the Supplementary Files section.

## Data Availability

Please address requests for materials to the corresponding authors.

## Abbreviations

ACSL4: Acyl-CoA synthetase long-chain family member 4
ALDH3A2: Aldehyde dehydrogenase 3A2
APOE: Apolipoprotein E
APOE3Ch: APOE Christchurch variant
AoD: Age of death
AoO: Age of onset
AUC: Area under the curve
BMI: Body mass index
BMP: Bis(monoacylglycero)phosphate
CE: Cholesteryl ester
Cer: Ceramide
cPLA2: Cytosolic phospholipase A2
dPCR: Digital PCR
DGAT2: Diacylglycerol acyltransferase 2
FAD: Familial Alzheimer’s disease
FC: Free cholesterol
GFAP: Glial fibrillary acidic protein
GO: Gene Ontology
IBA1: Ionized calcium-binding adapter molecule 1
IF: Immunofluorescence
MBOAT1: Membrane-bound O-acyltransferase 1
MFSD2A: Major facilitator superfamily domain-containing protein 2A
MCI: Mild cognitive impairment
PC: Phosphatidylcholine
PE: Phosphatidylethanolamine
PG: Phosphatidylglycerol
PI: Propidium iodide / Phosphatidylinositol (context-dependent)
PLTP: Phospholipid transfer protein
PMI: Postmortem interval
PRKD1: Protein kinase D1
PPM1L: Protein phosphatase 1L
PS: Phosphatidylserine
PSEN1: Presenilin-1
ROC: Receiver operating characteristic
SAD: Sporadic Alzheimer’s disease
SL: Sphingolipids
SM: Sphingomyelin
SMPD2: Sphingomyelin phosphodiesterase 2
SMPD3: Sphingomyelin phosphodiesterase 3
snRNA-seq: Single-nucleus RNA sequencing
SREBP2: Sterol regulatory element-binding protein 2
SVZ: Subventricular zone
TG: Triacylglycerol
TLC: Thin-layer chromatography
UGT8: UDP-galactose ceramide galactosyltransferase
VIM: Vimentin
WM: White matter
